# Not Carbon s–p Hybridization, but Coordination Number Determines C−H and C−C Bond Length

**DOI:** 10.1002/chem.202004653

**Published:** 2021-03-03

**Authors:** Pascal Vermeeren, Willem‐Jan van Zeist, Trevor A. Hamlin, Célia Fonseca Guerra, F. Matthias Bickelhaupt

**Affiliations:** ^1^ Department of Theoretical Chemistry Amsterdam Institute of, Molecular and Life Sciences (AIMMS) Amsterdam Center for Multiscale, Modeling (ACMM) Vrije Universiteit Amsterdam De Boelelaan 1083 1081 HV Amsterdam The Netherlands; ^2^ Leiden Institute of Chemistry, Gorlaeus Laboratories Leiden University Einsteinweg 55 2333 CC Leiden The Netherlands; ^3^ Institute for Molecules and Materials (IMM) Radboud University Heyendaalseweg 135 6525 AJ Nijmegen The Netherlands

**Keywords:** activation strain model, bonding analysis, density functional calculations, hybridization theory, Pauli repulsion

## Abstract

A fundamental and ubiquitous phenomenon in chemistry is the contraction of both C−H and C−C bonds as the carbon atoms involved vary, in s–p hybridization, along sp^3^ to sp^2^ to sp. Our quantum chemical bonding analyses based on Kohn–Sham molecular orbital theory show that the generally accepted rationale behind this trend is incorrect. Inspection of the molecular orbitals and their corresponding orbital overlaps reveals that the above‐mentioned shortening in C−H and C−C bonds is not determined by an increasing amount of *s*‐character at the carbon atom in these bonds. Instead, we establish that this structural trend is caused by a diminishing steric (Pauli) repulsion between substituents around the pertinent carbon atom, as the coordination number decreases along sp^3^ to sp^2^ to sp.

## Graphical abstract

**A paradigm shift**! Our quantum chemical analyses reveal that, for example, C−H bonds along ethane, ethylene, and acetylene, do not contract due to the changing s–p hybridization of the pertinent carbon atom from sp^3^ to sp^2^ to sp but, instead, because the steric congestion around this carbon atom decreases as the coordination number goes from 4 to 3 to 2.

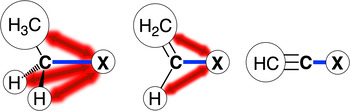

The geometrical properties of organic (and inorganic) molecules are, in general, explained using hybridization theory, which was introduced by Linus Pauling in 1931.[^1^, ^2^] A case in point is the fundamental and ubiquitous phenomenon in chemistry that C−H and C−C bonds contract as the carbon atoms involved vary, in s–p hybridization, along sp^3^ to sp^2^ to sp. Archetypal examples are the C−H and C−C bonds in ethane, ethene, ethyne and propane, propene, propyne, respectively. Hybridization theory ascribes the shortening of C−H and C−C bond lengths along sp^3^ to sp^2^ to sp to the increasing percentage of *s*‐character of the hybrid orbital at the pertinent carbon, as *s*‐orbitals are more contracted to the nucleus than *p*‐orbitals, thus giving rise to an optimal bond overlap at a shorter interatomic distance.[^3^, ^4^] This model is generally accepted and appears in most (physical) organic chemistry textbooks.[^5^, ^6^, ^7^, ^8^]

Herein, we show that the above standard model is incorrect. Through detailed quantum chemical bonding analyses of a series of representative, archetypal model systems (Figure 1), we have been able to reveal that the above‐mentioned shortening in C−H and C−C bonds is *not* related to an increasing amount of *s*‐character at the carbon atom in these bonds. Instead, we find that a diminishing steric (Pauli) repulsion between substituents around the carbon atom constitutes the physical mechanism behind the universal trend in molecular structure, as the number of substituents around the pertinent carbon atom decreases from 4 to 3 to 2 along sp^3^ to sp^2^ to sp hybridization, respectively. Our findings are based on the analysis of the C−H and C−C bonding mechanisms in a systematic series of model systems featuring sp^3^‐, sp^2^‐, and sp‐hybridized C−H and C−C bonds in saturated and unsaturated hydrocarbons (Figure 1), using the quantitative molecular orbital (MO) model contained in Kohn–Sham density functional theory (DFT)[^9^, ^10^, ^11^] at BP86/TZ2P[^12^, ^13^, ^14^] in combination with a matching canonical energy decomposition analysis (EDA) as implemented in the ADF program.[^15^, ^16^] Our findings are both, novel to the extent that they are paradigm‐changing and also suitably consistent with the well‐known role of steric repulsion in other contexts of molecular structure, such as, the stereochemical arrangement of substituents around a central atom or the dependence of bond distances on the steric bulk around the bond in question.[^17^, ^18^, ^19^, ^20^, ^21^, ^22^, ^23^, ^24^]

**Figure 1:**
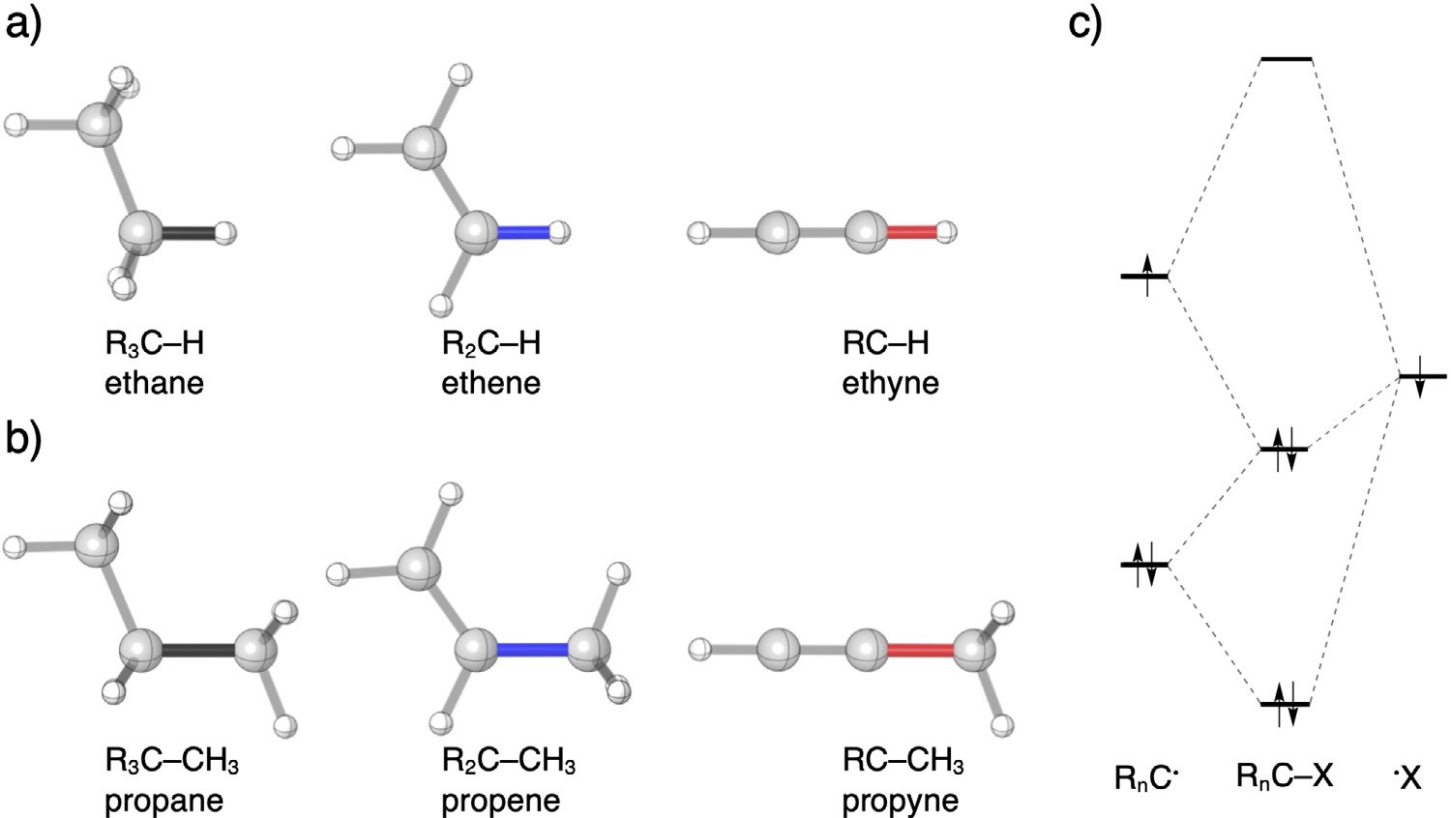
a) sp^3^‐, sp^2^‐, and sp‐hybridized C−H bonds of ethane, ethene, and ethyne, and b) sp^3^‐, sp^2^‐, and sp‐hybridized C−C bonds of propane, propene, and propyne, where the bond of interest is shown in black (sp^3^), blue (sp^2^), and red (sp). c) Schematic molecular orbital diagram of the formation of a generic R_n_C−X electron‐pair bond and interaction with a closed‐shell orbital that leads to (steric) Pauli repulsion.

Not unexpectedly, our DFT computations reproduce the aforementioned trend of a shortening of the C−H and C−C bond lengths as we go along sp^3^ to sp^2^ to sp hybridization of the carbon atom involved in such bonds (Table 1). The C−H bond length decreases along ethane (R_3_C−H, 1.099 Å), ethene (R_2_C−H, 1.091 Å), and ethyne (RC−H, 1.070 Å) while the corresponding C−C single bond lengths decrease along propane (R_3_C−CH_3_, 1.533 Å), propene (R_2_C−CH_3_, 1.500 Å), and propyne (RC−CH_3_, 1.456 Å). Note that in all cases, bond shortening correlates with bond strengthening as reflected by the increase in bond dissociation energy (BDE; Δ*E* = −Δ*E*_BDE_) along sp^3^ to sp^2^ to sp hybridization. In order to analyze the origin of the trend in bond strengths in more detail, we decompose the bond energy Δ*E* according to the activation strain model (ASM) of reactivity [Eq. 1]:[^25^, ^26^, ^27^, ^28^]

(1) $$\Delta E=\Delta E{}_{\mathrm{strain}}+\Delta E{}_{\mathrm{int}}$$

**Table 1:** Bond lengths (in Å) and energy decomposition analysis (in kcal mol^−1^) of the sp^3^‐, sp^2^‐, and sp‐hybridized C−H and C−C bonds in their equilibrium geometries.^[a]^

System^[b]^			Bond length	Δ*E*	Δ*E*_strain_	Δ*E*_int_	Δ*E*_Pauli_	Δ*V*_elstat_	Δ*E*_oi_
ethane	R_3_C−H	sp^3^	1.099	−106.8	7.2	−114.0	90.2	−62.8	−141.4
ethene	R_2_C−H	sp^2^	1.091	−115.8	3.1	−118.9	85.4	−60.1	−144.1
ethyne	RC−H	sp	1.070	−140.0	0.1	−140.1	40.4	−41.3	−139.1
propane	R_3_C−CH_3_	sp^3^	1.533	−89.3	18.3	−107.6	215.0	−138.9	−183.7
propene	R_2_C−CH_3_	sp^2^	1.500	−102.2	13.3	−115.5	221.4	−142.8	−194.2
propyne	RC−CH_3_	sp	1.456	−130.7	8.7	−139.4	171.2	−121.9	−188.8

[a] Computed at BP86/TZ2P level of theory. [b] R_3_C− = (H_3_C)H_2_C−; R_2_C− = (H_2_C)HC−; RC− = HCC−. See Figure 1 for the structures of the studied systems.

Here, the strain energy Δ*E*_strain_ is the penalty that needs to be paid for deforming the fragments from their equilibrium structure to the geometry they adopt at the equilibrium C−X (X = H, CH_3_) bond length. On the other hand, the interaction energy Δ*E*_int_ accounts for all mutual interactions that occur between the deformed fragments.

In all cases, the magnitude and trend in C−H and C−C bond dissociation energies appear to be determined by the interaction energies Δ*E*_int_. The strain energy Δ*E*_strain_ has only little influence on the calculated bond energy Δ*E* and does not affect the overall trend in relative bond strengths. They originate from the fact that, upon the formation of a new C−H or C−C bond, the other substituents around a carbon atom involved in the new bond bend away in order to reduce the otherwise even more destabilizing steric (Pauli) repulsion. This destabilizing effect is more pronounced when more substituents are around the carbon. Thus, Δ*E*_strain_ is most destabilizing for ethane (R_3_C‐H) and propane (R_3_C‐CH_3_) in which the intrinsically planar R_3_C^.^ radical undergoes pyramidalization.^[29]^ The geometrical deformations of the sterically less crowded R_2_C^.^ and RC^.^ radical fragments in, for example, ethene (R_2_C‐H) and ethyne (RC‐H) are less severe and, therefore, lead to lower strain energies.

In order to pinpoint the differences between the interaction energies, we have analyzed the various C−H and C−C bonds in great detail by decomposing the Δ*E*_int_ into three physically meaningful terms using the canonical energy decomposition analysis (EDA) scheme [Eq. 2]:^[9]^

(2) $$\Delta E{}_{\mathrm{int}}=\Delta V{}_{\mathrm{elstat}}+\Delta E{}_{\mathrm{Pauli}}+\Delta E{}_{\mathrm{oi}}$$

In Eq. (2), Δ*V*_elstat_ is the classical electrostatic interaction between the unperturbed charge distributions of the (deformed) reactants. The Pauli repulsion Δ*E*_Pauli_ comprises the destabilizing interaction between occupied orbitals due to the Pauli exclusion principle and is an excellent descriptor of steric repulsion. Finally, the orbital interaction Δ*E*_oi_ includes the formation of the electron‐pair bond between two singly occupied molecular orbitals (SOMOs) and orbital relaxation (i.e., charge transfer and polarization).

Note that the decomposed interaction energy terms depicted in Table 1 strongly depend on the C−H and C−C bond distance. Therefore, the differences between these energy terms along the hybridization series must be interpreted with special precaution because they emerge not only from the original variation in the intrinsic bonding properties but also from the concomitant geometrical relaxation which affects the original trends.[^25^, ^26^] In order to solely focus on the trend in the intrinsic bonding properties of our model systems, we have decomposed the interaction energy: (i) as a function of the C−H and C−C bond distance; while (ii) keeping R_n_C^.^ and H^.^ or CH_3_^.^ fragments fixed in the equilibrium geometry and valence electron configuration of the overall systems, i.e., R_n_C−H and R_n_C−CH_3_ (n = 1,2,3). The former ensures a consistent comparison of the energy terms at any bond distance whereas the latter prevents any other geometrical relaxation within the fragments to mask primary changes in the energy terms. Note that this measure guarantees that none of the primary effects in the interaction energy terms is absorbed into the strain term which remains constant.

Prior to discussing the decomposed interaction energy terms as a function of the bond length, we first examine the orbital overlap integrals corresponding to the C−H and C−C electron‐pair bonds (Figures 2 a and b; see Figure 1 c for molecular orbital diagram). The larger the overlap between the SOMOs of the two fragments, the more stabilizing the corresponding electron‐pair bonding orbital interaction.^[30]^ Thus, the point at which the SOMO–SOMO overlap reaches a maximum is often considered as an essential factor in determining the equilibrium bond length.[^1^, ^2^] These maxima follow a similar trend as the equilibrium bond lengths themselves, that is, as the fragments approach towards each other, the SOMO–SOMO overlap achieves its maximum earlier, at a longer bond distance, in the case of the sp^3^‐hybridized R_3_C^.^ than for the sp^2^‐hybridized R_2_C^.^ than for the sp‐hybridized RC^.^. This observation corresponds well with the spatial extent of the different hybridized SOMOs, which, in line with the current rationale, steadily decreases from the sp^3^‐hybridized R_3_C^.^ to the sp‐hybridized RC^.^ (Figure 2 c), in other words, the SOMO of R_3_C^.^ is closer to the grey vertical line than the SOMO of RC^⋅^. This can also be seen in the zoom‐in of Figure 2 d: if one approaches the carbon nucleus from infinity, the orbital function of R_3_C^.^ reaches the value of 0.05 au earlier, i.e., further away from the carbon nucleus, compared to the R_2_C^.^ and RC^.^ analogues. In addition, R_3_C^.^ also has a smaller orbital amplitude close to the carbon nucleus compared to R_2_C^.^ and RC^.^ (Figure 2 d), giving rise to less orbital overlap for the former as seen in Figures 2 a and 2b.

**Figure 2:**
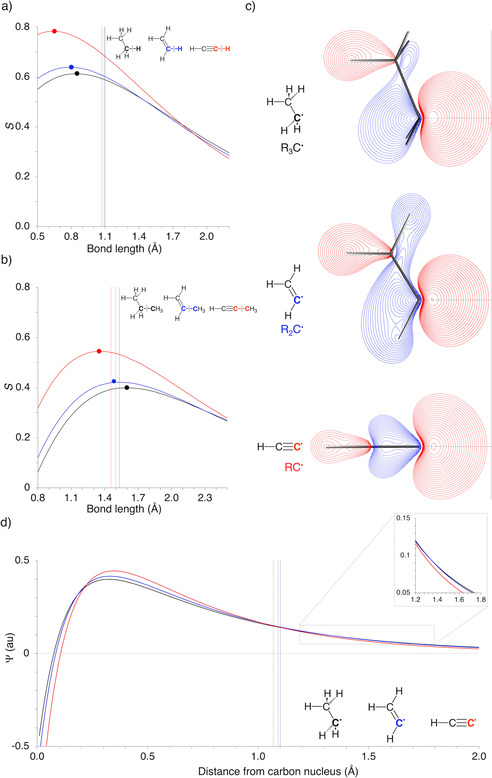
SOMO‐SOMO overlaps (*S*) of various hybridized a) C−H, and b) C−C bonds, where the maximum orbital overlap is indicated by a dot and the equilibrium bond length is indicated by a vertical line. c) Contour plots of the various hybridized SOMOs, where the gray vertical line indicates the maximum spatial extent of the sp^3^‐hybridized SOMO. All contour plots contain 25 contours from 0.05–0.5 au. d) Orbital function of the hybridized SOMOs along the investigated C−H bond (horizontal dashed gray line of Figure 2 c), where the equilibrium C−H bond lengths are indicated by a vertical line, and zoom‐in of the orbital function at the maximum spatial extent of the SOMOs in Figure 2 c.

Note, however, two striking phenomena: (i) all equilibrium C−H, and C−C, bond distances differ significantly from the distance at which the bond overlaps achieve their maximum, C−H bonds are in fact all longer; and (ii) the contraction of C−H, and also C−C, bonds as the carbon atoms involved vary, in s–p hybridization, along sp^3^ to sp^2^ to sp, is significantly smaller than the variation in the distance at which the corresponding bond overlaps achieve their maximum (see vertical lines and dots in Figures 2 a and b). Thus, despite the fact that the positions of the maximum SOMO‐SOMO overlap display the expected trends, other physical mechanisms are crucial for achieving the actual equilibrium bond distances.

Our energy decomposition analysis as a function of the C−X (X = H, CH_3_) distance, shows that, in contrast to present‐day textbook knowledge, the orbital interactions Δ*E*_oi_ are not responsible for the stronger and shorter sp‐hybridized C−H and C−C bond (Figures 3 a and b). The interaction energy Δ*E*_int_ follows the trend discussed earlier, i.e., bonds involving sp^3^‐hybridized carbon atoms are weaker and have a less stabilizing Δ*E*_int_ than their sp‐hybridized analogs. Strikingly, the orbital interactions Δ*E*_oi_, however, show an opposite behavior: from sp^3^‐ to sp^2^‐ to sp‐hybridized carbon atom in C−X bonds, the Δ*E*_oi_ curves become *decreasingly* stabilizing, although the difference between sp^3^ and sp^2^ hybridization is only marginal. This trend stems from the shrinking contribution of orbital relaxation which relieves the Pauli repulsion, especially at shorter C−X distances at which closed‐shell–closed‐shell repulsion becomes very large. Thus, if it were for the orbital interactions alone, C−X bonds would become longer, not shorter, along sp^3^, sp^2^, and sp hybridization of the carbon atom.^[31]^ The electrostatic attraction Δ*V*_elstat_ follows a similar trend along the series as Δ*E*_oi_, i.e., the curves become decreasingly stabilizing and shallow along sp^3^, sp^2^, and sp and, therefore, also favor elongation of the C−X bond distance along this series.

**Figure 3:**
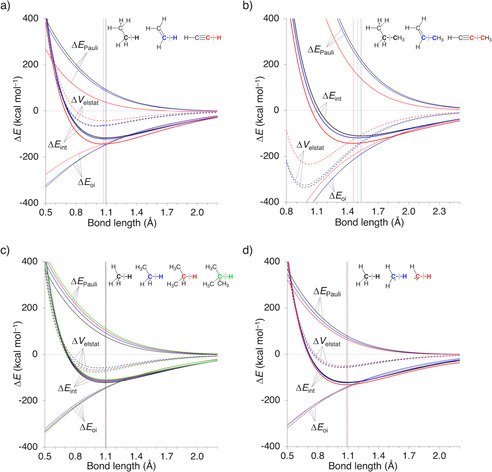
Energy decomposition analysis of a) C−H and b) C−C bonds. c) sp^3^‐hybridized C−H bonds with an increasing number of bulky substituents, and d) sp^3^‐hybridized C−H bonds with a decreasing number of substituents, where the equilibrium bond lengths are indicated by a vertical line.

We now identify Δ*E*_Pauli_ as the decisive factor in determining the equilibrium bond length because the only difference between the sp^3^‐ and sp^2^‐hybridized C−X bonds lies in this repulsive term, which is less destabilizing for the latter. This difference allows the fragments to approach each other more closely, leading to shorter bond distances. Continuing to the bonds involving an sp‐hybridized carbon atom, there is a remarkably large drop in Pauli repulsion Δ*E*_Pauli_. This effect partly compensates the weakening of Δ*E*_oi_ and, especially for C−C, also of Δ*V*_elstat_. It is therefore the change in Δ*E*_Pauli_ that determines the longer bond lengths in sp^3^‐hybridized C−H and C−C bonds compared to their sp^2^‐ and sp‐hybridized analogs. We recall that this phenomenon is also displayed in the EDA terms corresponding to the equilibrium geometries (Table 1). As shown, a highly destabilizing Δ*E*_Pauli_ induces an elongation of the C−X bond, which, in turn, reduces all EDA terms, including the Δ*E*_Pauli_. Nevertheless, the Δ*E*_Pauli_ of the longer sp^3^‐hybridized C−X bond is more destabilizing than the less hybridized counterparts, indicating that it is this term that governs the observed lengthening of the C−X bond.

The relation between the Pauli repulsion and the number of sterically hindering substituents becomes even more evident when, in numerical experiments, we explicitly change the size and number of substituents (see Table S1). Increasing substituent size leads to a longer sp^3^‐hybridized C−H bond (Figure 3 c; H_3_C−H: 1.096 Å to (H_3_C)_3_C−H: 1.104 Å), whereas a decreasing number of substituents makes this bond shorter (Figure 3 d; H_3_C−H: 1.096 Å to HC^..^−H: 1.085 Å). Inspection of the corresponding energy plots shows that the modulation of the equilibrium bond length, and consequently bond strength, is again caused by the Δ*E*_Pauli_. The Δ*V*_elstat_ and Δ*E*_oi_, on the contrary, do not vary that much along the series. More precisely, they counteract the observed trend in Δ*E*_int_.

Importantly, our analyses also shed light on the nature, especially the orbital energy, of the σ*‐orbital of the sp^*n*^‐hybridized R_n_C−X bonds (n = 3, 2, 1; X = H, alkyl, halogen, etc.), which is of direct relevance for understanding various types of reactions and supramolecular aggregates featuring these bonds.[^32^, ^33^, ^34^] We find that the σ*‐orbital of R_n_C−X bonds becomes increasingly more stabilized, on going from sp^3^ to sp^2^ to sp carbon centers, again, due to a reduction in the number of substituents around the pertinent carbon atom. The σ*‐orbital of the sp^*n*^‐hybridized R_n_C−H bond lowers in energy along n = 3, 2, 1, from 1.7 eV for R_3_C−H to 1.4 eV for R_2_C−H to 1.0 eV for RC−H, respectively, because the R_n_C^.^ SOMO becomes gradually more stabilized. This behavior can be ascribed to two phenomena: (i) the R_n_C^.^ SOMO is R_n_−C_2*s*_ antibonding, which reduces as R_n_ decreases from n = 3 to 2 to 1, due to less orbital overlap; (ii) the R_n_C^.^ SOMO is also R_n_−C_2*p*_ bonding and its bonding capability, i.e., orbital overlap, becomes stronger as R_n_ can align better with C_2*p*_ along this series (see Supporting Information Discussion 1 and Figure S1 for a detailed molecular orbital analysis).

To conclude, we have shown that, in contrast to the present‐day paradigm, the contraction of C−H and C−C bond lengths on going from sp^3^ to sp^2^ to sp carbon centers, originates from a diminished Pauli repulsion, the magnitude of which is directly related to the steric proximity between the substituents around the pertinent carbon atom. The orbital interaction, which was up to this point seen as the driving force, shows behavior that counteracts the observed trend in bond strength and, consequently, is not responsible for the decreasing bond length.

## Conflict of interest

The authors declare no conflict of interest.

## Supporting information

As a service to our authors and readers, this journal provides supporting information supplied by the authors. Such materials are peer reviewed and may be re‐organized for online delivery, but are not copy‐edited or typeset. Technical support issues arising from supporting information (other than missing files) should be addressed to the authors.

Supplementary
