# Supplementary material for: Not Carbon s–p Hybridization, but Coordination Number Determines C−H and C−C Bond Length
Source: Chemistry. 2021 Mar 3;27(24):7074–9. doi: 10.1002/chem.202004653 (PMC8248318; doi:10.1002/chem.202004653)
Supplement: Supplementary file 1 — Supplementary [file CHEM-27-7074-s001.pdf]

Chemistry–A European Journal

Supporting Information

Not Carbon s–p Hybridization, but Coordination Number Determines C–H and C–C Bond Length

Pascal Vermeeren,[a] Willem-Jan van Zeist,[a] Trevor A. Hamlin,[a] Célia Fonseca Guerra,[a, b] and F. Matthias Bickelhaupt*[a, c]

Content

Computational Details

Supporting Discussion S1: Stability of the $\sigma^*_{C\text{–}H}$ orbitals with different C hybridization.

Figure S1: a) The formation of a C–H bond involving a sp^3- (black), sp^2- (blue), and sp-hybridized (red) carbon atom. The construction of b) $R_3C^•$ ($\langle R_3|C_{2s}\rangle = 0.73$ and $\langle R_3|C_{2p}\rangle = 0.21$), c) $R_2C^•$ ($\langle R_2|C_{2s}\rangle = 0.69$ and $\langle R_2|C_{2p}\rangle = 0.23$), and d) $RC^•$ ($\langle R|C_{2s}\rangle = 0.60$ and $\langle R|C_{2p}\rangle = 0.35$). The orbital energies are displayed in eV and calculated using an unrestricted formalism at BP86/TZ2P. See Supporting Discussion 1 for a detailed molecular orbital analysis.

Table S1: Bond lengths (in Å) and energy terms (in kcal mol^{-1}) of the sp^3-, sp^2-, and sp-hybridized C–H and C–C bonds with increasing and decreasing steric bulk in their equilibrium geometries.

Table S2: Orbital energies (in eV) and orbital overlap integrals of the sp^3-, sp^2-, and sp-hybridized simple H_nC–H systems (n = 3, 2, 1).

Table S3: Cartesian coordinates (in Å) and ADF total bonding energies (E, in kcal mol^{-1}) of all molecular species used in this study, computed at BP86/TZ2P.

Computational Details

All calculations were performed using the Amsterdam Density Functional (ADF) program.[1] The MOs were expanded using a large uncontracted set of Slater-type orbitals (STOs) containing diffuse functions: TZ2P.[2] The TZ2P is of triple-ζ quality and has been augmented with two sets of polarization functions: 2p and 3d on hydrogen, 3d and 4f on carbon. The core-shell of carbon (1s) was treated by the frozen core approximation. An auxiliary set of s, p, d, f and g STOs was used to fit the molecular density and to represent the Coulomb and exchange potentials accurately in each SCF cycle. Equilibrium structures were checked using vibrational analysis; all structures described have zero imaginary frequencies (see Table S1 for cartesian coordinates).[3] Energies and gradients were computed using the BP86 functional,[4] which is one of the three best DFT functionals regarding the accuracy of geometries.[5] Energy decomposition analyses (EDA) of C–H and C–C electron-pair bonds as a function of bond distance were carried out using the PyFrag program.[6] Structures in Figure 1 were rendered using CYLview.[7]

---

[1] a) G. te Velde, F. M. Bickelhaupt, E. J. Baerends, C. Fonseca Guerra, S. J. A. van Gisbergen, J. G. Snijders, T. Ziegler, *J. Comput. Chem.* **2001**, *22*, 931; b) C. Fonseca Guerra, J. G. Snijders, G. te Velde, E. J. Baerends, *Theor. Chem. Acc.* **1998**, *99*, 391; c) ADF, SCM Theoretical Chemistry, Vrije Universiteit: Amsterdam (Netherlands). http://www.scm.com.

[2] a) E. van Lenthe, E. J. Baerends, *J. Comput. Chem.* **2003**, *24*, 1142; b) M. Franchini, P. H. T. Philipsen, E. van Lenthe, L. Visscher, *J. Chem. Theory Comput.* **2014**, *10*, 1994.

[3] a) A. Bérces, R. M. Dickson, L. Fan, H. Jacobsen, D. Swerhone, T. Ziegler, *Comput. Phys. Commun.* **1997**, *100*, 247; b) H. Jacobsen, A. Bérces, D. P. Swerhone, T. Ziegler, *Comput. Phys. Commun.* **1997**, *100*, 263; c) Wolff, S. K. *Int. J. Quantum Chem.* **2005**, *104***,** 645.

[4] a) J. P. Perdew, *Phys. Rev. B* **1986**, *33*, 8822; b) A. D. Becke, *Phys. Rev. A* **1988**, *38*, 3098.

[5] M. Swart, J. G. Snijders, *Theor. Chem. Acc.* **2003**, *110*, 34.

[6] X. Sun, T. M. Soini, J. Poater, T. A. Hamlin, F. M. Bickelhaupt, *J. Comp. Chem.* **2019**, *40*, 2227.

[7] C. Y. Legault, CYLview, Unversité de Sherbrooke, Sherbrooke, QC (Canada), **2009**.

Supporting Discussion S1: Stability of the $\sigma^*_{C–H}$ orbitals with different C hybridization.

As observed in our previous work,[33] the energy of the σ^*-orbital of an sp^n-hybridized R_nC–H bond (n = 3, 2, 1) is stabilized when the hybridization of the pertinent carbon atom goes from sp^3 to sp^2 to sp, namely, from 1.7 eV for R_3C–H to 1.4 eV for R_2C–H to 1.0 eV for RC–H (see Figure S1). This $\sigma^*_{C–H}$ orbital is the antibonding combination between the alkyl $R_nC^•$ (n = 1, 2, 3) and hydrogen $H^•$ SOMOs. Because the hydrogen SOMO remains unchanged for all three sp^n-hybridized bonds, the observed drop in $\sigma^*_{C–H}$ orbital energy solely originates from the increasingly more stable $R_nC^•$ SOMO, along this series, namely, $R_3C^•$ = –5.5 eV, $R_2C^•$ = –6.3 eV, $RC^•$ = –9.8 eV.

The stabilization of the $R_nC^•$ SOMO, on going from n = 3 to 2 to 1, arises from the change in orbital overlap between the R_n orbital (left side of the molecular diagrams in Figure S1b-d) and the atomic orbitals (AOs) of the pertinent carbon atom (right side of the molecular diagrams in Figure S1b-d). The carbon atom is in a $2s^12p^3$ valence electron configuration, and for that reason only has one *s* and one *p* atomic orbital which can interact with the R_n orbital and construct the $R_nC^•$ SOMO. The $R_nC^•$ SOMO is R_n–C_{2s} antibonding, meaning that the R_n and C_{2s} orbitals have an out-of-phase overlap. As a result, the larger the R_n–C_{2s} overlap, the more the $R_nC^•$ SOMO becomes destabilized (increase in energy). Furthermore, the $R_nC^•$ SOMO is also R_n–C_{2p} bonding, because the R_n and C_{2s} orbitals have an in-phase overlap. In this case, a larger R_n–C_{2p} overlap will lead to a more stabilized $R_nC^•$ SOMO (decrease in energy).

The overlap between the antibonding R_n and C_{2s} orbitals reduces when the sp^n-hybridized $R_nC^•$ SOMO goes from n = 3 to 2 to 1 ($\langle R_3|C_{2s}\rangle$: 0.73, $\langle R_2|C_{2s}\rangle$: 0.69, $\langle R|C_{2s}\rangle$: 0.60), which, in turn, leads to an increasingly less destabilized $R_nC^•$ SOMO. This is mainly due to the reducing number of lobes of the R_n orbital, *i.e.*, number of substituents around the involved carbon atom, along the series. In other words, for the construction of the sp^3-hybridized $R_3C^•$ SOMO, the C_{2s} overlaps with three R_n lobes which decrease to only one R_n lobe for the sp-hybridized $RC^•$ SOMO, leading to less R_n–C_{2s} antibonding character for the latter and, consequently, a more stabilized sp-hybridized $R_nC^•$ SOMO. In addition, the reducing number of R_n lobes also results in a more collinear alignment between R_n and the C_{2p} AO of the pertinent carbon atom, amplifying the bonding R_n–C_{2p} character. As the number of R_n lobes reduces, going from an sp^3- to an sp-hybridized $R_nC^•$ SOMO, the R_n lobe points more towards the C_{2p} orbital, resulting in better R_n–C_{2p} orbital overlap ($\langle R_3|C_{2p}\rangle$: 0.21, $\langle R_2|C_{2p}\rangle$: 0.23, $\langle R|C_{2p}\rangle$: 0.35) and, as a response, a more stabilized $R_nC^•$ SOMO.

This rationale behind the trend in $\sigma^*_{C–H}$ orbital energy holds for all hybridized systems, even the simplest radical hydrocarbons (see Table S2). The $\sigma^*_{C–H}$ orbital of the sp^3-hybridized H_3C–H bond is less stable than the $\sigma^*_{C–H}$ orbital of the sp-hybridized $HC^{\bullet\bullet}$–H analog, due to the prior discussed more dominant H_n–C_{2s} antibonding interaction and a weaker H_n–C_{2p} bonding interaction for the former. Furthermore, we can extrapolate the herein presented rationale to the σ*-orbital of all types of C–X bonds. The $\sigma^*_{C–X}$ orbital of a bond containing an sp^3-hybridized carbon will always be less stable than the sp^2- or sp-hybridized analog, with equal substituent X, because only the alkyl SOMO varies along these bonds. With the help of this clarification, we can qualitatively predict and explain the main conclusion of Ref. 33. The activation of an arylic C–X bond occurs with a consistently lower reaction barrier than the activation of the aliphatic counterpart due to a lower $\sigma^*_{C–X}$ acceptor orbital. This can, as shown here, be explained solely based on the hybridization of the carbon atom participating in the C–X bond.

In conclusion, the $\sigma^*_{C–X}$ orbital of an sp^n-hybridized R_nC–X bond (n = 1, 2, 3; X = H, alkyl, halogen, etc.) becomes stabilized (decrease in energy) as the number of substituents decreases, along n = 3, 2 and 1. The reason is that the $R_nC^{\bullet}$ SOMO lowers in energy along this series because of a reduced number of R_n orbital lobes. The carbon *2s* atomic orbital overlaps with fewer R_n lobes going from sp^3 to sp^2 to sp, respectively, thereby reducing the antibonding destabilization of the $R_nC^{\bullet}$ SOMO. Additionally, the R_n lobes become more aligned with the C_{2p} atomic orbital when going from sp^3 to sp, which enhances the orbital overlap and stabilizes the SOMO with even a greater extent. This rationale can be used to quantitatively predict chemical reactivity and non-covalent bond strength.

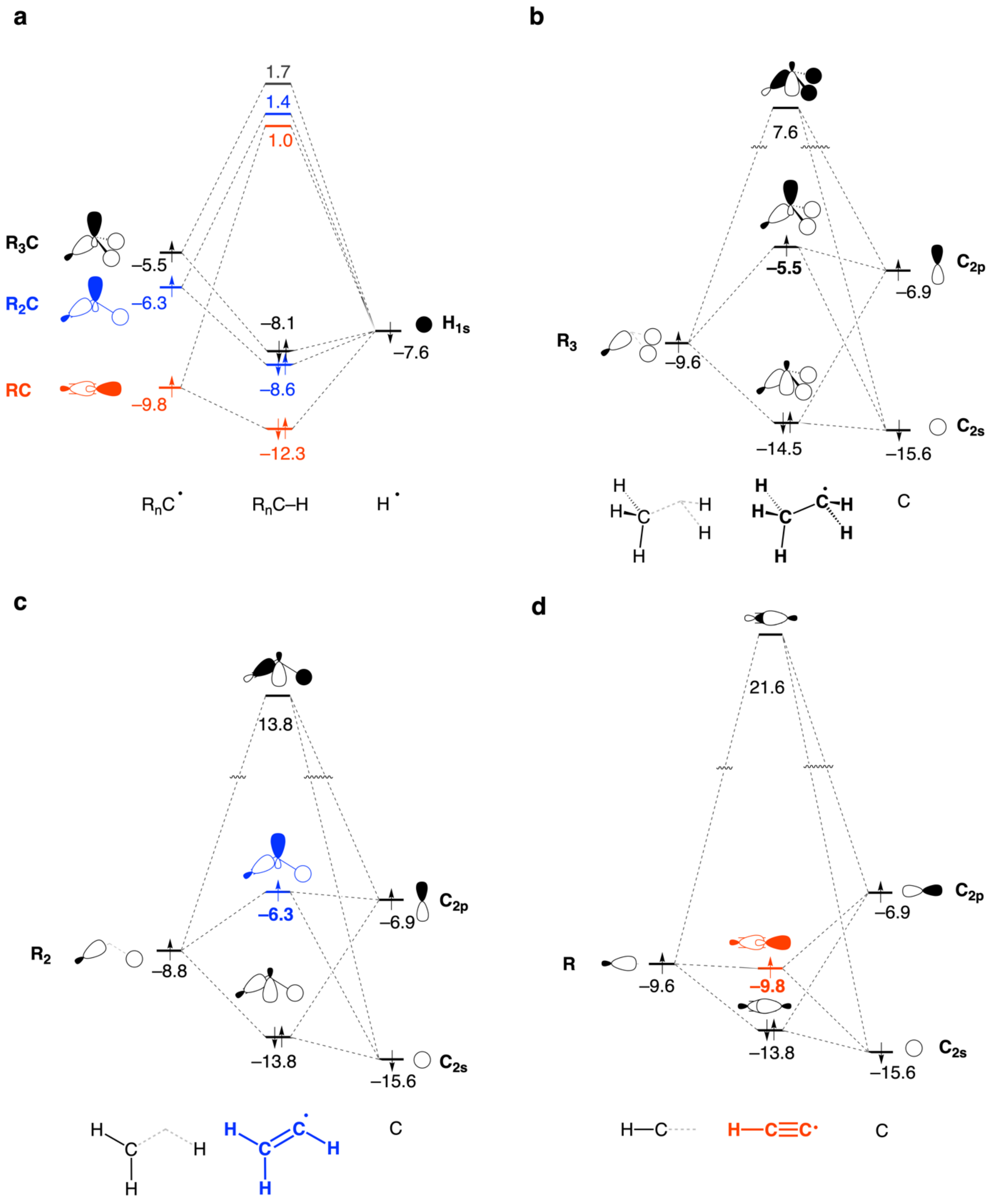

Figure S1: a) The formation of a C–H bond involving a sp^3- (black), sp^2- (blue), and sp-hybridized (red) carbon atom. The construction of b) $R_3C^•$ ($\langle R_3|C_{2s}\rangle$ = 0.73 and $\langle R_3|C_{2p}\rangle$ = 0.21), c) $R_2C^•$ ($\langle R_2|C_{2s}\rangle$ = 0.69 and $\langle R_2|C_{2p}\rangle$ = 0.23), and d) $RC^•$ ($\langle R|C_{2s}\rangle$ = 0.60 and $\langle R|C_{2p}\rangle$ = 0.35). The orbital energies are displayed in eV and calculated using an unrestricted formalism at BP86/TZ2P. See Supporting Discussion 1 for a detailed molecular orbital analysis.

Table S1: Bond lengths (in Å) and energy terms (in kcal mol^{-1}) of the sp^3-, sp^2-, and sp-hybridized C–H and C–C bonds with increasing and decreasing steric bulk in their equilibrium geometries.[a]

System	Bond Length	ΔE	ΔE_{strain}	ΔE_{int}	ΔV_{elstat}	ΔE_{Pauli}	ΔE_{oi}
H_3C–H	1.096	–111.9	7.1	–119.0	–55.8	76.0	–139.2
$(H_3C)H_2C$–H	1.099	–106.8	7.2	–114.0	–62.8	90.2	–141.4
$(H_3C)_2HC$–H	1.102	–102.7	7.3	–110.0	–68.0	99.9	–141.8
$(H_3C)_3C$–H	1.104	–99.4	7.2	–106.6	–71.8	107.3	–142.0
$H_2C^{\bullet}$–H	1.086	–117.7	2.1	–119.8	–52.4	69.4	–136.8
$HC^{\bullet\bullet}$–H	1.085	–115.5	11.7	–127.2	–48.1	61.8	–141.0
H_3C–CH_3	1.532	–93.1	18.3	–111.4	–127.5	194.8	–178.7
$(H_3C)_3C$–CH_3	1.540	–82.3	18.6	–100.9	–151.2	235.0	–184.7
$(H_3C)_3C$– [illegible]	1.591	–63.1	25.8	–88.9	–157.5	244.0	–175.4

[a] Computed at BP86/TZ2P.

Table S2: Orbital energies (in eV) and orbital overlap integrals of the sp^3-, sp^2-, and sp-hybridized simple H_nC–H systems (n = 3, 2, 1).[a,b]

System	Point group	Fragment		$\varepsilon_{\sigma^*\text{HnC–H}}$	$\varepsilon_{\text{HnC}\bullet\text{ SOMO}}$	$\langle H_n \mid C_{2s}\rangle$	$\langle H_n \mid C_{2p}\rangle$
H_3C–H	T_d	$H_3C^{\bullet}$	sp	0.43	–6.0	0.81	0.25
H_2C–$H^{\bullet}$	D_{3h}	$H_2C^{\bullet\bullet}$	sp	0.42	–6.9	0.73	0.33
HC–$H^{\bullet\bullet}$[c]	$D_{\infty h}$ (linear)	$HC^{\bullet\bullet\bullet}$	sp	0.30	–9.6	0.59	0.50

[a] Computed at BP86/TZ2P. [b] Energies are calculated using an unrestricted formalism. [c] Linear $CH_2^{\bullet\bullet}$ is not an energy minimum, but a 2nd order saddle point.

Table S3: Cartesian coordinates (in Å) and ADF total bonding energies (E, in kcal mol^{-1}) of all molecular species used in this study, computed at BP86/TZ2P.

C_2H_6 **[-923.00]**

C	0.000000	0.000000	0.000000
C	1.531788	0.000009	0.000000
H	1.932782	0.511614	0.886200
H	-0.401106	-0.511614	0.886200
H	-0.401106	-0.511614	-0.886200
H	1.932800	-1.023286	0.000000
H	1.932782	0.511614	-0.886200
H	-0.638611	0.895643	0.000000

C_2H_4 **[-729.15]**

C	0.000000	0.666244	0.000000
C	0.000000	-0.666244	0.000000
H	0.928188	1.239428	0.000000
H	-0.928188	1.239428	0.000000
H	-0.928188	-1.239428	0.000000
H	0.928188	-1.239428	0.000000

C_2H_2 **[-523.62]**

H	0.000000	0.000000	-0.172595
C	0.000000	0.000000	0.897171
C	0.000000	0.000000	2.102829
H	0.000000	0.000000	3.172595

C_3H_8 **[-1298.62]**

C	0.000000	0.000000	-0.024218
H	0.000000	-0.879867	0.638584
H	0.000000	0.879867	0.638584
C	1.277623	0.000000	-0.870571
H	2.180048	0.000000	-0.243380
H	1.321814	0.887015	-1.520471
H	1.321814	-0.887015	-1.520471
C	-1.277623	0.000000	-0.870571
H	-2.180048	0.000000	-0.243380
H	-1.321814	0.887015	-1.520471
H	-1.321814	-0.887015	-1.520471

C_3H_6 **[-1108.62]**

C	0.004800	0.001464	0.000000
C	1.505155	0.001907	0.000000
H	1.909374	1.022720	0.000000
H	1.900247	-0.526399	0.881684
H	1.900247	-0.526399	-0.881684
H	-0.474694	-0.982909	0.000000
C	-0.768226	1.090686	0.000000
H	-1.855804	1.019190	0.000000
H	-0.338409	2.095069	0.000000

C_3H_4 **[-907.24]**

C	0.000000	0.000000	-0.230062
C	0.000000	0.000000	1.226207
H	0.513034	-0.888601	1.620469
H	0.513034	0.888601	1.620469
H	-1.026068	0.000000	1.620469
C	0.000000	0.000000	-1.439724
H	0.000000	0.000000	-2.507885

CH_4 **[-548.73]**

C	0.000000	0.000000	0.000000
H	0.632707	0.632707	-0.632707
H	-0.632707	0.632707	0.632707
H	0.632707	-0.632707	0.632707
H	-0.632707	-0.632707	-0.632707

C_4H_{10} **[-1674.82]**

C	0.000000	0.000000	-0.030135
H	0.000000	0.000000	1.073565
C	-0.730669	1.265555	-0.499973
H	-0.757488	1.312008	-1.600600
H	-1.769716	1.286634	-0.139986
H	-0.229400	2.175936	-0.139986
C	1.461337	0.000000	-0.499973
H	1.999116	0.889302	-0.139986
H	1.514976	0.000000	-1.600600
H	1.999116	-0.889302	-0.139986
C	-0.730669	-1.265555	-0.499973
H	-1.769716	-1.286634	-0.139986
H	-0.757488	-1.312008	-1.600600
H	-0.229400	-2.175936	-0.139986

$CH_3^{\bullet}$ (D_{3h} Trigonal planar) **[-414.94]**

C	0.000000	0.000000	0.000000
H	1.085851	0.000000	0.000000
H	-0.542926	0.940375	0.000000
H	-0.542926	-0.940375	0.000000

$CH_3^{\bullet}$ (T_d Tetrahedral) **[-391.88]**

C	0.000000	0.000000	0.000000
H	0.632706	-0.632706	0.632706
H	0.632706	0.632706	-0.632706
H	-0.632706	0.632706	0.632706

$CH_2^{\bullet\bullet}$ ($D_{\infty h}$ Linear) **[-271.48]**

C	0.000000	0.000000	0.000000
H	0.000000	0.000000	1.073713
H	0.000000	0.000000	-1.073713

$CH_2^{\bullet\bullet}$ (D_{3h} Trigonal) **[-273.02]**

C	0.000000	0.000000	0.000000
H	0.000000	0.940418	0.542951
H	0.000000	-0.940418	0.542951

C_5H_{12} **[-2050.81]**

C	0.000000	0.000000	-0.018141
C	0.725794	-1.257111	0.495047
H	0.741036	-1.283512	1.595359
H	0.227379	-2.173078	0.142600
H	1.768251	-1.283455	0.142600
C	0.725794	1.257111	0.495047
H	1.768251	1.283455	0.142600
H	0.741036	1.283512	1.595359
H	0.227379	2.173078	0.142600
C	-1.451587	0.000000	0.495047
H	-1.995630	-0.889623	0.142600
H	-1.482072	0.000000	1.595359
H	-1.995630	0.889623	0.142600
C	0.000000	0.000000	-1.557818
H	-0.513623	0.889621	-1.953280
H	1.027246	0.000000	-1.953280
H	-0.513623	-0.889621	-1.953280

C_8H_{18} **[-3170.12]**

C	0.795274	-0.000028	-0.000212
C	-0.795274	0.000028	-0.000212
C	1.354959	0.842367	1.167276
C	1.354997	0.589875	-1.313479
C	1.354997	-1.432280	0.145631
C	-1.354959	-0.842367	1.167276
C	-1.354997	-0.589875	-1.313479
C	-1.354997	1.432280	0.145631
H	2.448467	0.728005	1.215255
H	0.949183	0.526582	2.139172
H	1.142077	1.912753	1.040366
H	2.448505	0.688589	-1.238387
H	0.949232	1.589468	-1.525928
H	1.142125	-0.055208	-2.177022
H	2.448505	-1.416593	0.022582
H	0.949234	-2.116143	-0.613737
H	1.142183	-1.857464	1.136130
H	-1.142077	-1.912753	1.040366
H	-2.448467	-0.728005	1.215255
H	-0.949183	-0.526582	2.139172
H	-1.142125	0.055208	-2.177022
H	-2.448505	-0.688589	-1.238387
H	-0.949232	-1.589468	-1.525928
H	-1.142183	1.857464	1.136130
H	-2.448505	1.416593	0.022582
H	-0.949234	2.116143	-0.613737
